# Evaluation of cholesterol transformation abilities and probiotic properties of *Bacteroides dorei* YGMCC0564

**DOI:** 10.3389/fmicb.2023.1279996

**Published:** 2023-11-09

**Authors:** Zhili He, Tinghui Wang, Shichang Zhang, Kuojiang Shi, Fan Wang, Yanzhao Li, Chanqing Lin, Jianguo Chen

**Affiliations:** ^1^Beijing YuGen Pharmaceutical Co., Ltd., Beijing, China; ^2^Beijing Hotgen Biotechnology Inc., Beijing, China

**Keywords:** *B. dorei* YGMCC0564, cholesterol, next-generation probiotics, bile salt, short-chain fatty acids

## Abstract

Hypercholesterolemia, a risk factor for cardiovascular disease (CVD), often requires therapeutic agents with varying degrees of side effects. This has created a need for safe and natural alternatives such as medications or functional foods that can improve lipid metabolism and reduce cholesterol levels. In recent years, Next-generation probiotics (NGPs) have recently emerged as a potential solution, offering distinct mechanisms compared to traditional probiotics. Among the NGPs, *Bacteroides*, a dominant bacterial genus in the human gut, has gained significant attention due to its prevalence, ability to break down plant polysaccharides, and production of short-chain fatty acids (SCFAs). Recent evidence has demonstrated that *Bacteroides* effectively reduces cholesterol levels, prevents obesity, and lowers the risk of CVD. However, research on *Bacteroides* is currently limited to a few species, leaving rooms for exploration of the beneficial functions of different species in this genus. In this study, we isolated 66 *Bacteroides* strains, including 9 distinct species, from healthy adults’ fecal samples. By comparing their ability to assimilate cholesterol, we found that the transformation ability was not specific to any particular species. Notably, *Bacteroides dorei* YGMCC0564 revealed superior cholesterol-lowering capabilities and bile salt hydrolase (BSH) activity *in vitro*, surpassing that of *Lactobacillus* GG (LGG). YGMCC0564 exhibited favorable probiotic characteristics, including high survival rate *in vitro* simulation of gastrointestinal digestion, excellent adhesion ability, susceptibility to antibiotics, absence of hemolysis or virulence genes, and substantial production of SCFAs. The strain also demonstrated remarkable bile salt deconjugation activities and upregulation of the *BT_416* gene associated with cholesterol, providing insights into a possible molecular mechanism underlying its cholesterol-reducing activity. These findings establish YGMCC0564 as a promising NPG candidate for improving cardiovascular health.

## Background

CVD is a major global health issue and one of the leading causes of death worldwide (Virani et al., 2021). High levels of cholesterol in the blood, known as hypercholesterolemia, are considered a risk factor and a predictive marker for CVD, which can lead to the development of atherosclerosis, heart failure, or hypertension (Song et al., 2021). Currently, statins are the primary medications used to treat hypercholesterolemia. However, long-term use of these drugs has been associated with adverse effects such as diabetes, muscle pain, muscle weakness, and reduced kidney function (Ramkumar et al., 2016; Ferri and Corsini, 2020). Therefore, there is an urgent need to discover natural and safe medications or functional foods that can prevent and reduce blood lipid levels.

Traditional probiotics, such as Lactobacillus and Bifidobacterium, are mainly derived from fermented foods, breast milk or intestines. They have a long history of safe use and are usually consumed as food or dietary supplements (Singh and Natraj, 2021). Their mechanisms of action include modulating the host immune system, producing antimicrobial substances or essential molecules, but these mechanisms are often strain- and host-specific and lack molecular-level analysis. Next-generation probiotics are based on specific human gut commensals, and are screened and designed by genome sequencing, synthetic biology, and culture genomics, to target and treat specific diseases or populations. Their mechanism of action involves the metabolic network of the gut microbiota, and they modulate intestinal molecular targets to intervene in host physiology and disease. Their mechanism of action can be revealed by multi-omics technologies (Chang et al., 2019). For example, *Akkermansia muciniphila* metabolites SCFAs (Kim et al., 2021), cell membrane phospholipids a15:0-i15:0 PE (Bae et al., 2022), and outer membrane protein Amuc_1100 (Plovier et al., 2017) can improve intestinal permeability by activating cannabinoid 1 receptors and enhancing tight junctions, increasing immunocytokine secretion and restoring intestinal flora balance, thus improving the physical, immune and biological barrier functions of the gut and enhancing GI function. Therefore, the main difference between traditional and next-generation probiotics is the source, screening method, mechanism of action and application target. Traditional probiotics are randomly selected from nature and have general and non-specific effects, while next-generation probiotics are based on human intestinal commensals and have targeted and personalized effects.

Traditional probiotics, specifically *Lactobacillus* and *Bifidobacterium*, have been extensively studied for their beneficial effects in lowering cholesterol levels *in vitro*, animal experiments, and clinical trials (Suez et al., 2019; Wang et al., 2022). For example, a double-blind, randomized, parallel controlled study showed that consuming yogurt with *Lactobacillus reuteri* NCIMB 30242 led to reductions in triglyceride (TG), high-density lipoprotein (HDL), and low-density Lipoprotein (LDL) by approximately 4.8, 8.9, and 6.0% respectively (Jones et al., 2012). A meta-analysis involving 38 clinics up until 2022 also confirmed the effectiveness of probiotics in reducing TG, total cholesterol (TC), and LDL, providing strong support for probiotic supplementation as an effective intervention to improve lipid profiles (Zarezadeh et al., 2023). In recent years, next-generation sequencing has greatly expanded our knowledge of microorganisms that have potential benefits for the host. These microorganisms, known as NGPs, mainly belong to the *Akkermansia*, *Bacteroides*, and *Faecalibacterium*. NGPs are defined as “microbes which have not been used to date as agents to promote health, and which are more likely to be delivered under a drug regulatory framework” (O'Toole et al., 2017). Numerous recent studies have confirmed the cholesterol-lowering effects of NGPs. Specifically, significant reductions in TC, TG and LDL were observed in hypercholesterolaemic mice fed with *Akkermansia muciniphila* (Katiraei et al., 2020). *F. prausnitzii* is a member of the genus *Faecalibacterium*, which has probiotic potential and is considered one of the most important bacterial indicators of gut health. Compared to high fat diet (HFD) mice, mice treated with *F. prausnitzii* had lower levels of liver fat, triglycerides and cholesterol (Munukka et al., 2017). Furthermore, a microbial cholesterol sulfotransferase gene (*Bt_0416*) from *Bacteroides thetaiotaomicrom* DSM2079 has been identified, which encodes an enzyme that transforms cholesterol into cholesterol sulfate. This discovery revealed a novel mechanism by which symbiotic gut microorganisms degrade cholesterol (Le et al., 2022). A review in 2022 provided a comprehensive summary of gut microbiome-mediated mechanisms for reducing cholesterol levels (Jia et al., 2022): (i) cholesterol adsorption and absorption by bacteria; (ii) conjugated secondary bile acids further hydrolyzed by microbial bile salt hydrolases; and (iii) cholesterol reduction through the production of cholesterol-lowering metabolites such as short-chain fatty acids (SCFAs) and secondary bile acids (BA); (iv) transformation of cholesterol to coprostanol; (v) conversion of cholesterol to sulfonated cholesterol. While the first three mechanisms are the main ways traditional probiotics work, the latter two are more specific and effective pathways for cholesterol reduction in NGPs. Based on these findings, we believe that NGPs have great potential for the prevention and treatment of CVD.

*Bacteroides* is now of great interest as an important source of NGPs, which is commonly found in the intestines, mouth, respiratory tract, and reproductive tract of humans and animals. It makes up 5–18% of the total human microbiota and is more abundant than well-known bacteria like *Lactobacillus* and *Bifidobacterium* (Tan et al., 2019). *Bacteroides* plays a crucial role in the intestinal environment and affects human physiology. *Bacteroides* has a strong ability to break down plant-derived carbohydrates that the body cannot easily absorb. They convert these carbohydrates into absorbable substances and produce short-chain fatty acids, which are utilized by other gut microbes (You et al., 2023). Thus, *Bacteroides* serves as an important supplier of nutrients in the gut. Moreover, previous studies (Ridlon et al., 2006; Wahlström et al., 2016) have demonstrated that Bacteroides exhibit bile salt hydrolase activity not only in the small intestine but also in the large intestine. This enzymatic activity enables the direct conversion of cholesterol within the intestinal lumen (Gérard et al., 2007; Le et al., 2022), leading to a significant improvement in cholesterol metabolism. An early classic study investigated the relationship between gut microbiota and obesity and showed that fecal macrobiota transplantation (FMT)-induced obesity was reduced in mice housed with mice containing mainly *Bacteroides*, suggesting an anti-obesity effect of *Bacteroides* (Turnbaugh et al., 2006). Weight loss was associated with increased abundance of the *Bacteroidetes* (including *Bacteroides* spp. and *Prevotella* spp.) (Dong et al., 2022). *B. uniformis* CECT7771 was shown to significantly reduce body weight gain and liver steatosis in mice fed a high-fat diet (Gauffin Cano et al., 2012; Fabersani et al., 2021). Simultaneous interventions with *B. vulgatus* and *B. dorei* reduced atherosclerotic lesion formation in mice. Bioactive substances secreted by *B. dorei* directly stimulated farnesol X receptor (FXR) in intestinal epithelial cells, thereby ameliorating the obesity (Zhang et al., 2015). Despite these findings, research on *Bacteroides* is still limited, and there is much to explore in terms of mechanisms and the development of new beneficial NGPs.

In the present study, a group of *Bacteroides* strains, consisting of 9 different species, was isolated from fecal samples of healthy adults. The cholesterol-transforming ability of *Bacteroides* strains was compared, revealing that the capability is not species-specific. *Bacteroides* strains with high cholesterol degradation and positive BSH activity were then screened for their tolerance to simulate gastrointestinal fluid and safety. Eventually, a promising strain called *B. dorei* YGMCC0564 was identified. Finally, we investigated the potential cholesterol-lowering mechanism of YGMCC0564. These findings provide a theoretical basis for the development of live biotherapeutics for lipid-lowering applications and offer a promising candidate for NGPs to improve CVD.

## Materials and methods

### Bacterial isolation and purification

Fresh fecal samples were exclusively collected from a cohort of 35 healthy women. The volunteers met the following inclusion criteria: (1) age between 28 and 36 years, non-pregnant, and leading a healthy lifestyle with a balanced diet; (2) no use of antimicrobial drugs or any other medications that can affect the intestinal micro-ecology in the last 6 months; (3) no smoking, alcohol consumption, or engagement in other unhealthy habits; (4) absence of gastrointestinal tumors, polyps, or inflammatory bowel disease (IBD) and other digestive disorders; (5) no major diseases like diabetes, heart disease, hypertension, or malignant tumors. This study protocol was approved by the Ethics Committee of the Chinese Centre for Disease Control and Prevention (Beijing, China, agreement no. 2023-001) and all volunteers provided written informed consent prior to participation. Samples were collected using 10 mL sterile sampling tubes, stored at 4°C in an ice box, and transported to the laboratory within 2 h for bacterial isolation. Fecal samples were serially diluted 10^−1^ to 10^−9^fold in reduced Phosphate buffer saline (PBS) (0.1 g feces per ml PBS), and 100 μL of each dilution was plated directly onto non-selective media YCFA agar. Plates were incubated under anaerobic condition in an atmosphere of 85% N_2_, 10% CO_2_ and 5% H_2_ at 37°C for 2–3 d. Single colonies were picked and streaked onto new plates to obtain single clones. All colonies were identified by MALDI-TOF (Matrix-assisted laser desorption/ionization-time of flight) and stored at −80°C in a glycerol suspension (25%, v/v) containing 0.1% cysteine.

Colonies were identified using MALDI–TOF MS EXS2000 (Zybio Inc., Hongqing, China). Each deposit was covered with 2 mL of a matrix solution (saturated α-cyano acid-4- hydroxycinnamic in 50% acetonitrile and 2.5% trifluoroacetic acid). MS data were analyzed using MDT Master (version 1.1). *E. coli* ATCC 25922 was used for mass calibration and optimization of instrument parameters to achieve a mean deviation in molecular weight of less than 300 ppm after correction. An isolate was then considered correctly identified at the species level if at least one of the colony spectra had a score ≥2.0 and another of the colony spectra had a score ≥1.7. If the species could not be accurately identified by MALDI-TOF after three attempts, the isolate was identified by 16S rRNA sequencing as previously described (Duncan et al., 2002). A threshold similarity value of >98.7% was chosen for species-level identification.

The evolutionary history was inferred using the Neighbor-Joining method (Saitou and Nei, 1987). The optimal tree is shown. The percentage of replicate trees in which the associated taxa clustered together in the bootstrap test (1,000 replicates) is shown next to the branches (Felsenstein, 1985). Evolutionary distances were calculated using the Kimura 2-parameter method (Kimura, 1980) and are expressed in terms of the number of base substitutions per site. This analysis included 13 nucleotide sequences. All ambiguous positions were removed for each sequence pair (pairwise deletion option). Evolutionary analyses were performed in MEGA11 (Tamura et al., 2021).

### Strains and culture conditions

The *Bacteroides* strains used in this study were isolated from healthy adults. The *Bacteroides thetaiotaomicron* DSM2079 strain was obtained from the China Center of Industrial Culture Collection (CICC). *Lactobacillus rhamnosus* GG (LGG), a commercial probiotic strain, was also used as a reference strain.

Brain heart infusion supplemented (BHIS) medium preparation: BHI powder (Thermo Scientific^™^ Oxoid, UK) was dissolved in distilled water at 37 g/L, and heme chloride and vitamins were added to give a final concentration of 0.01 ‰ (*w/v*). BHIS plates were prepared on the above basis supplemented with 15 g/L agar, conjugated bile salt [0.05% Glycodeoxycholic acid, GDCA (*w/v*) (Macklin., China) or 0.15% Tauroursodeoxycholic acid, TDCA (*w/v*) (Macklin., China)]. BHIS-TC medium was prepared by adding of cholesterol-PEG-600 (Sigma, United States) to BHIS to a final concentration of 10 mg/mL.

### Cholesterol assimilation ability

Three passages of activated bacteria were transferred to fresh BHIS and incubated at 37°C for 20 h. 1 × 10^7^ CFU bacteria liquid was transferred to the BHIS-TC medium and incubated anaerobically at 37°C. Non-bacterial tubes were used as blank controls and all samples were set in triplicate. The fermented supernatant was collected after 24 h and the excess cholesterol in the supernatant was measured by using a cholesterol detection kit (Nanjing Jiancheng，China). Cholesterol conversion = (C-A)/C 100%, where A in the formula is the TC concentration of the supernatant after fermentation, and C is the TC concentration of the blank control medium.

### Bile salt hydrolase activity

The third generation of activated bacteria were inoculated into BHIS plates, BHIS-GDCA (containing 0.05% GDCA), or BHIS-TDCA (containing 0.15% TDCA), respectively. Plates were cultured for 48 h and images were collected. If a strain has the BSH activity, it will degrade the conjugated bile acid, causing the substrate to precipitate in the medium. BSH enzyme evaluation criteria: no precipitation (−), mild precipitation (+), extensive fog-like precipitation (++).

### Tolerance to simulation of gastrointestinal digestion *in vitro*

Took 3 g of pepsin (1:15,000 U, Shangon Bioted, China) dissolved in 1,000 mL aseptic physiological saline (0.9% w/v) to a final concentration of 3 g/L. The solution was adjusted to pH 3 with hydrochloric acid and filtered through a 0.22 μm sterile filter. Simulate colon liquid configuration: take 1 g of trypsin (1:250 U, Shangon Bioted, China) was dissolved in 1,000 mL of aseptic physiological saline (0.9% w/v) to a final concentration of 1 g/L. The solution was adjusted to pH 8 with 2 M NaOH and filtered through a 0.22 μm sterile filter. The activated bacterial liquid was used for gradient dilution and then incubated anaerobically on the plate for 24 h before counting. The remaining bacterial solution was centrifuged and resuspended in an equal volume of simulate gastric juice (pH 3.0). After anaerobic culture of 37°C for 2 h, the bacteria were collected for gradient dilution and viable counts were conducted. The remaining bacteria were then resuspended in an equal volume of simulate colonic fluid (pH 8.0) and incubated for 4 h, and the bacteria were collected for viable counts. Three replicates were set for each strain.

### Cell adhesion ability

Cell adhesion abilities were evaluated by auto-aggregation and hydrophobicity assays performed according to the method (Zhao et al., 2021) with some modifications. After incubation at 37°C for 18 h, *Bacteroides* strains in PBS were adjusted to an optical density of 0.60 at 600 nm (A_0_ or H_0_). Briefly, 4 mL of cell suspension of each strain was incubated at 20°C for 24 h, the absorbance of the 1 mL of the bacterial cell suspensions in the upper phase was measured as the value of OD600nm (A_1_). Auto-aggregation ability was calculated with the following formula: A (%) = (1 − A_1_/A_0_) × 100%.

1 mL of chloroform was added to 3 mL of cell suspension and was vortexed for 3 min. The aqueous phase was collected and measured at 600 nm (H_1_) after incubation at 37°C for 1 h. The value of hydrophobicity was calculated as follows: H (%) = (1 − H_1_/H_0_) × 100%.

### Hemolytic activity

Strains were inoculated on Columbia agar containing 5% sheep blood and the plates were incubated at 37°C for 48 h. After incubation, the hemolytic activity was evaluated and classified based on the lysis of red blood cells in the medium around the colonies. The clear zones around colonies (β-hemolysis) and no zones around colonies (γ-hemolysis) on Columbia blood agar plates. Only strains with γ-hemolysis are considered as safe.

### Antibiotic susceptibility

Strains were tested using the disk diffusion method according to the Clinical and Laboratory Standards Institute guidelines (Zhou et al., 2022). The nine antibiotic disks used in this study were Ampicillin (AMP, 10 μg), Ampicillin-sulbactam (AMS, 20 μg), Cefotetan (CTT, 30 μg), Imipenem (IMI, 10 μg), Tetracycline (TE, 30 μg), Moxifloxacin (MXF, 5 μg), Clindamycin (CD, 2 μg), Chloramphenicol (C, 30 μg) and Metronidazole (MTZ, 5 μg). The antibiotic disks were placed on BHIS plates spread by Bacteroides stain (1 × 10^8^ CFU/mL) and the inhibition zone diameter was measured in millimeters after incubation at 37°C for 48 h.

### Whole-genome sequencing

The whole genome sequence of YGMCC0564 was sequenced using the Illumina NovaSeq PE150 system. The ResFinder web tool, the Resistance Gene Identifier in the Comprehensive Antimicrobial Resistance Database (CARD) and the Virulence Factor Database (VFDB) were used to analyze the strains for antibiotic resistance genes and virulence factors.

### HPLC analysis of SCFAs

HPLC detection and quantification of SCFAs was conducted according to the method with minor modifications (Ahmed et al., 2019). Briefly, the HPLC determinations were performed as described in Supplementary Table S1.

### Quantitative determination of bile salt deconjugation ability

Strains were removed from the cold refrigerator and passaged three times for activation. The activated strains were inoculated into MRS or BHIS medium at 1% (*v/v*) for overnight culture. The concentration of each strain was adjusted to OD (600 nm) = 1.0, and then washed twice with 0.1 M HAc NaAc buffer (pH 5.0). After mixing 400 μ L of bacterial suspension and 400 μL of 20 mM of bile salt mixture (TDCA + GDCA), they were incubated in a 37°C water bath for 1 h. Control samples were prepared by mixing 400 μL of 20 mM bile salt mixture and 400 μL of HAc NaAc buffer. The reaction was terminated by adding 800 μL of 15% TCA (Macklin, China), and the supernatant of the mixture was collected at −80°C. The sample must be filtered through a 0.22 μm nylon membrane for HPLC analysis. Detection parameters were shown in Supplementary Table S2.

### Statistical analyses

All data are shown as Means ± SD. Samples were set up in triplicate and complete experiments were repeated independently more than twice. Statistical comparisons were performed with Student’s unpaired and paired *T* tests.

## Results

### Isolation and identification of the bacterial strains

**Figure 1:**
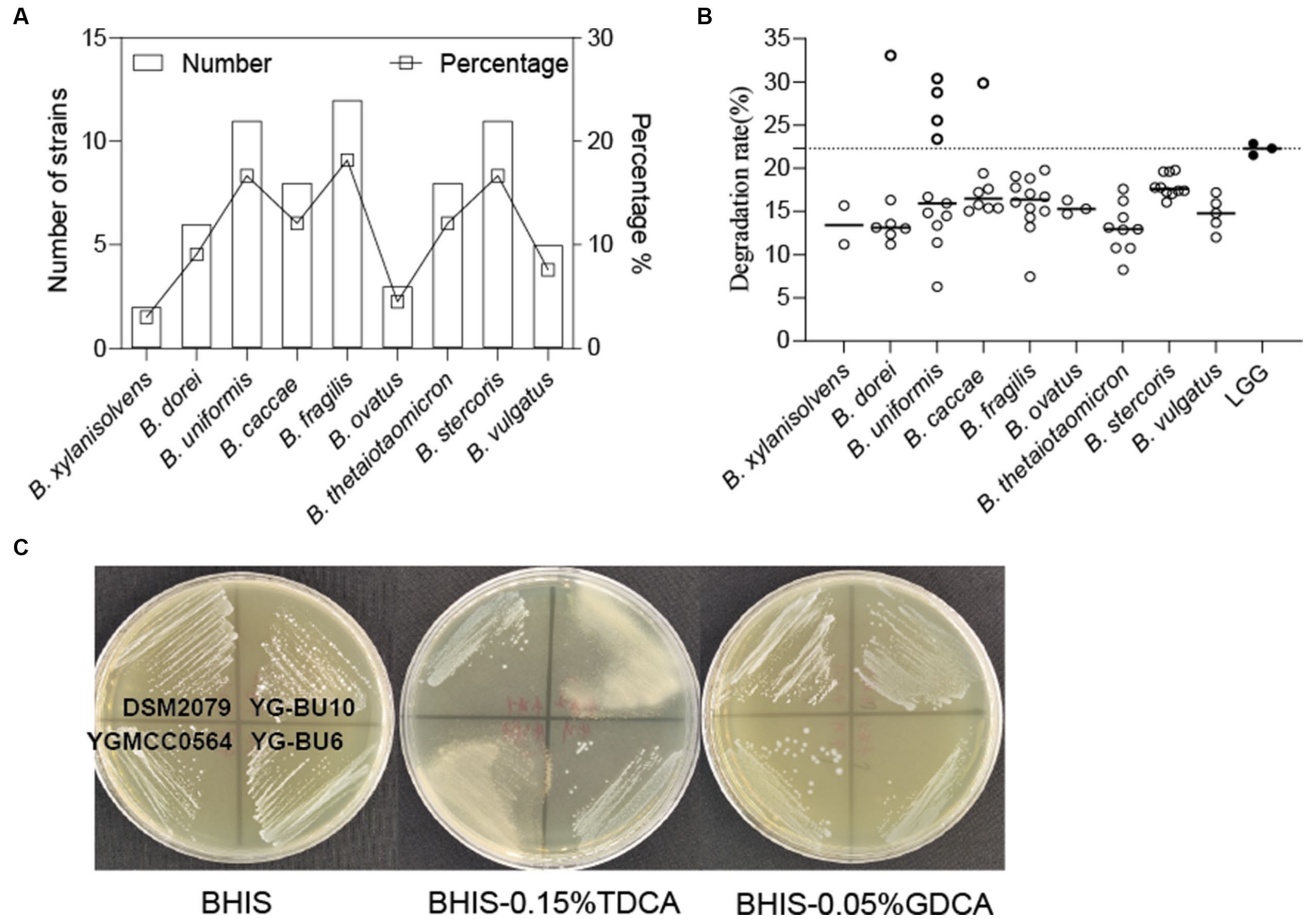
Assessment of the ability of the strains to assimilate cholesterol. **(A)** *Bacteroides* species distribution of 66 strains from 35 human fecal samples. **(B)** TC assimilation rates of different *Bacteroides* spp. Thicky circles represent strains with better transformation than LGG. Independent experiments were repeated twice. **(C)** Bile salt hydrolase activity of the strains.

Through MALDI-TOF MS analysis, we identified a total of 501 colonies from fecal samples of 35 healthy women, and successfully obtained 66 *Bacteroides* strains, which belonged to *B. fragilis* (18.2%), *B. uniformis* (16.7%), *B. stercoris* (16.7%), *B. thetaiotaomicron* (12.1%), *B. caccae* (12.1%), *B. dorei* (9.1%), *B. vulgatus* (7.6%), *B. ovatus* (4.5%), and *B. xylanisolvens* (3%) (Figure 1A).

### Cholesterol assimilation ability

As shown in Figure 1B and Table 1, most of the 66 strains belonging to nine different species had cholesterol removal rates ranging from 10 to 20%. It is noteworthy that several *Bacteroides* strains showed better cholesterol assimilation abilities than LGG, including one *B. dorei*, four *B. uniformis*, and one *B. caccae*.

**Table 1:** Cholesterol assimilation rate and BSH activity of *Bacteroides* strains.

Number	Name	TC%(Mean + SD)	TDCA	GDCA
DSM2079	*B. thetaiotaomicron*	13.01 ± 2.82	−	−
*LGG*	*Lactobacillus* GG	22.22 ± 9.73	−	−
**YG-BC8**	**B. caccae**	29.84 ± 2.42	++	−
**YG-BU10**	**B. uniformis**	30.38 ± 4.86	++	−
YG-BU6	*B. uniformis*	28.77 ± 3.26	−	−
**YG-BU7**	**B. uniformis**	25.54 ± 5.49	++	−
**YG-BU5**	**B. uniformis**	23.39 ± 3.70	++	−
YG-BU1	*B. uniformis*	14.84 ± 2.39	++	−
YG-BU4	*B. uniformis*	14.48 ± 1.38	++	−
YG-BU2	*B. uniformis*	13.38 ± 1.38	++	−
YG-BF10	*B. fragilis*	14.32 ± 2.07	++	−
**YGMCC0564**	**B. dorei**	33.07 ± 5.64	++	−
YG-BV4	*B. vulgatus*	13.14 ± 2.72	++	−
YG-BV3	*B. vulgatus*	11.18 ± 3.11	++	−
YG-BX2	*B. xylanisolvens*	11.18 ± 1.56	+	+

The meaning of the bold values indicates strain has both cholesterol assimilation rate >22.22% and BSH enzyme positive.

### Bile salt hydrolase activity

This precipitation is shown as a fog-like precipitate in Figure 1C. The BSH activity and cholesterol assimilation efficiencies of all the BSH-positive bacteria are listed in Table 1. Among the 66 strains, YG-BC8, YG-BU1, YGBU2, YG-BU4, YG-BU5, YG-BU7, YG-BU10, YG-BF10, YGMCC0564, YG-BV3, YG-BV4, and YG-BX2 were able to degrade TDCA and presented significant precipitation on the medium (++ or +). This precipitation is shown as a fog-like precipitate in Figure 1C. Among these, only YG-BX 2 was able to react with GDCA and produce precipitation (+), whereas LGG and DSM2079 showed no activity on either medium.

### The tolerance of the simulate gastrointestinal fluid

Normally, probiotics have to go through multiple pressures such as gastric acid and intestinal fluid to colonize the colon and perform their own function. To assess the probiotic properties, the tested strains were exposed to simulate gastric juice for 2 h, followed by simulate intestinal fluid for 4 h before calculating survival rates. According to Table 2, out of the five functional strains, three *B. uniformis* strains showed poor tolerance to simulate gastrointestinal fluid, particularly in the intestinal fluid environment, with a survival rate of 0%. *B. caccae* YG-BC8 was tolerant to simulate gastrointestinal fluid, but less than LGG and DSM2079. On the other hand, *B. dorei* YGMCC0564 demonstrated excellent tolerance, with a survival rate of 93.84% after exposure to gastrointestinal fluid. At the same time，genes encoding proteins that are involved in acid tolerance were identified in the YGMCC 0564 genome. These include genes such as dTDP-glucose 4,6-dehydratase (*rfbB*), 4-hydroxy-tetrahydrodipicolinate synthase (*dapA*), ATP dependent intracellular protein (*ClpP*), and F0F1-ATPases (such as *atpC*, *atpD*, *atpG*) (Hamon et al., 2014; Sun et al., 2022; Zhao et al., 2023). These acid tolerance-related genes are likely responsible for YGMCC0564’s excellent acid resistance.

**Table 2:** The survival rate of simulate gastrointestinal fluid of strains.

Strain		0 h in PBS	2 h in simulate gastric juice (pH 3.0)		4 h in simulate intestinal juice (pH 8.0)	
		Log CFU/mL	Log CFU/mL	Survival rate (2 h/0 h %)	Log CFU/mL	Survival rate (4 h/0 h %)
DSM2079	*B. thetaiotaomicron*	10.73 ± 0.02	9.64 ± 0.25	89.81	7.9 ± 0.16	73.63
LGG	*Lactobacillus* GG	8.8 ± 0.05	8.81 ± 0.05	100.11	8.14 ± 0.15	92.50
YGMCC0564	*B. dorei*	10.22 ± 0.05	10.43 ± 0.08	102.05	9.59 ± 0.12	93.84
YG-BU10	*B. uniformis*	9.39 ± 0.05	4.49 ± 0.25	47.82	0 ± 0	0.00
YG-BU5	*B. uniformis*	9.73 ± 0.26	6.12 ± 0.12	62.90	0 ± 0	0.00
YG-BU7	*B. uniformis*	9.01 ± 0.09	0 ± 0	0.00	0 ± 0	0.00
YG-BC8	*B. caccae*	8.97 ± 0.07	6.14 ± 0.02	68.45	5.44 ± 0.4	58.42

### Morphology analysis and identification of YGMCC0564

**Figure 2:**
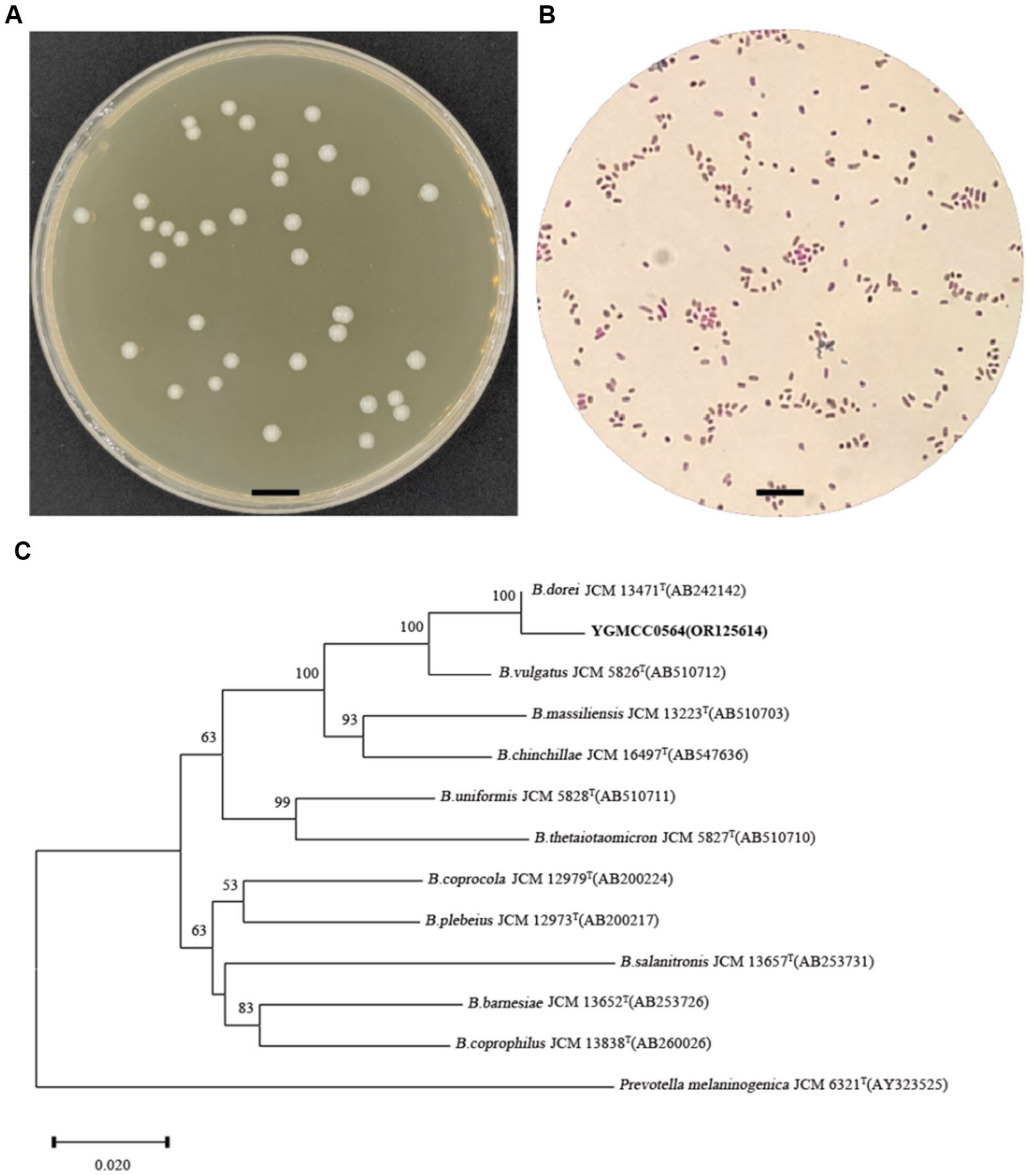
Morphology analysis and identification of YGMCC0564. **(A)** Colony morphology of YGMCC0564 on BHIS plate. **(B)** Gram staining morphology. **(C)** Neighbor-joining tree based on the 16S rRNA gene sequences of YGMCC0564 and the related species in the genus *Bacteroides*. *Prevotella melaninogenica* JCM 6321^T^ was used as out-group. The bar represents 0.02 substitutions per nucleotide position.

On BHIS agar, the YGMCC0564 strain displayed a sticky, white, and mucoid colony morphology, with colonies ranging from 1 to 2 mm in diameter (Figure 2A). The cells of YGMCC0564 were gram-negative, non-motile, and had a short-rod-shaped structure with regular ends, approximately 0.7–0.8 μm in width and 1–3 μm in length (Figure 2B). Based on the results of 16S rRNA sequence analysis results (Figure 2C), YGMCC0564 is phylogenetically most related to *B. dorei* JCM 13471^T^, which can support the classification of YGMCC0564 as *B. dorei*, and its 16S rRNA gene sequence has been submitted to NCBI with the accession number of OR125614.

### Genome map of YGMCC 0564

**Figure 3:**
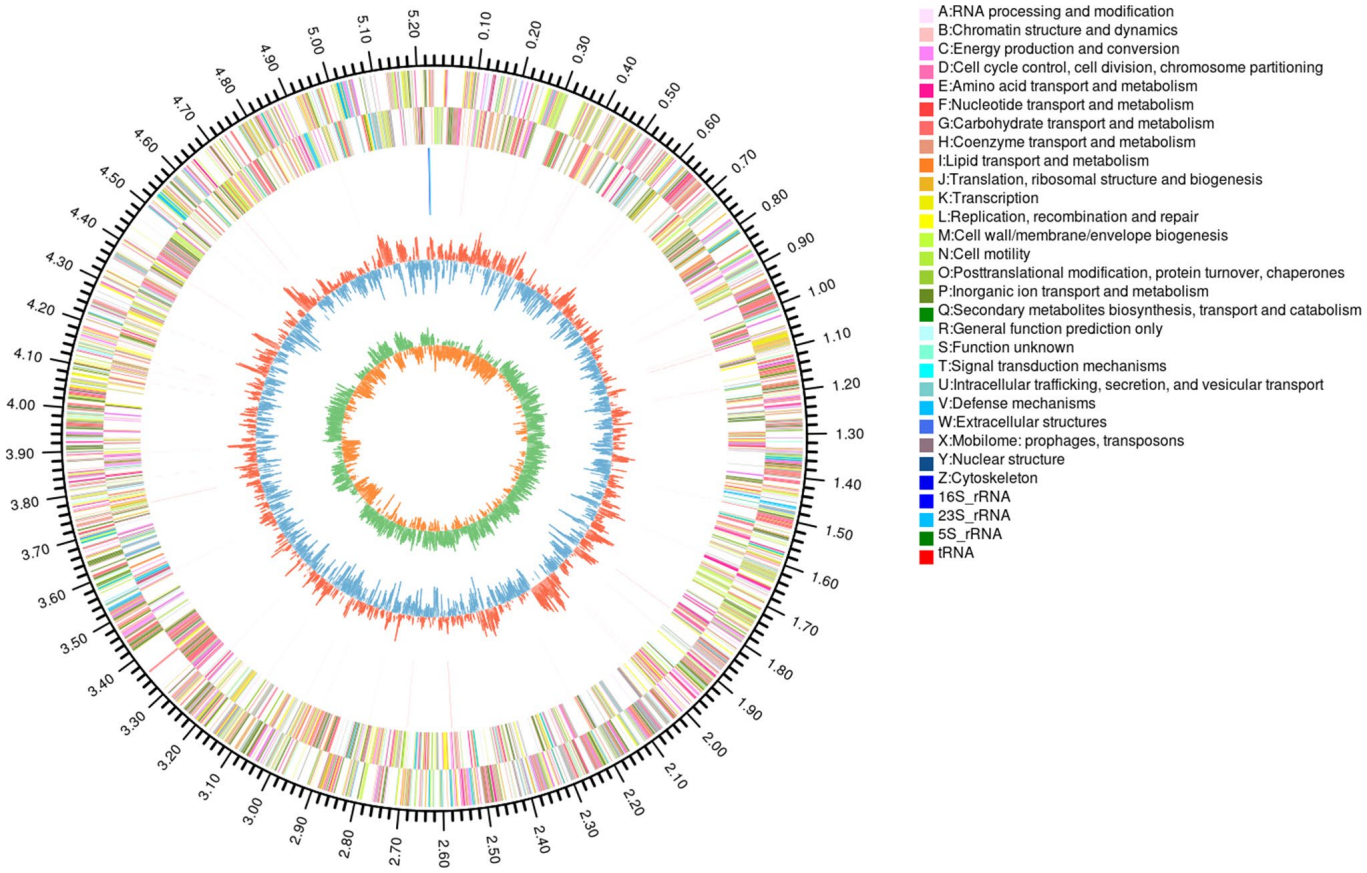
Circular genome map of *B. dorei* YGMCC 0564. From outside to center: the first circle represented genome size; the 2nd and 3rd circles represented CDS of the forward and reverse strand; the 4th represented rRNA and tRNA; the fifth represented GC content.

The complete genome sequence of YGMCC0564 contained a length of 5,240,181 bp with a GC content of 41.92% (Figure 3). Among the predicted genes, there were 4,294 genes with a coding ratio of 98.34%, including 4,223 protein coding genes and 71 RNA genes (Table 3).

**Table 3:** *B. dorei* YGMCC 0564 genome features.

Attribute	Values
Genome size (bp)	5,240,181
GC content (%)	41.92
Total genes	4,294
CDS (protein)	4,223
tRNA genes	68
rRNA genes	3

### Adhesion property and hemolytic test

**Figure 4:**
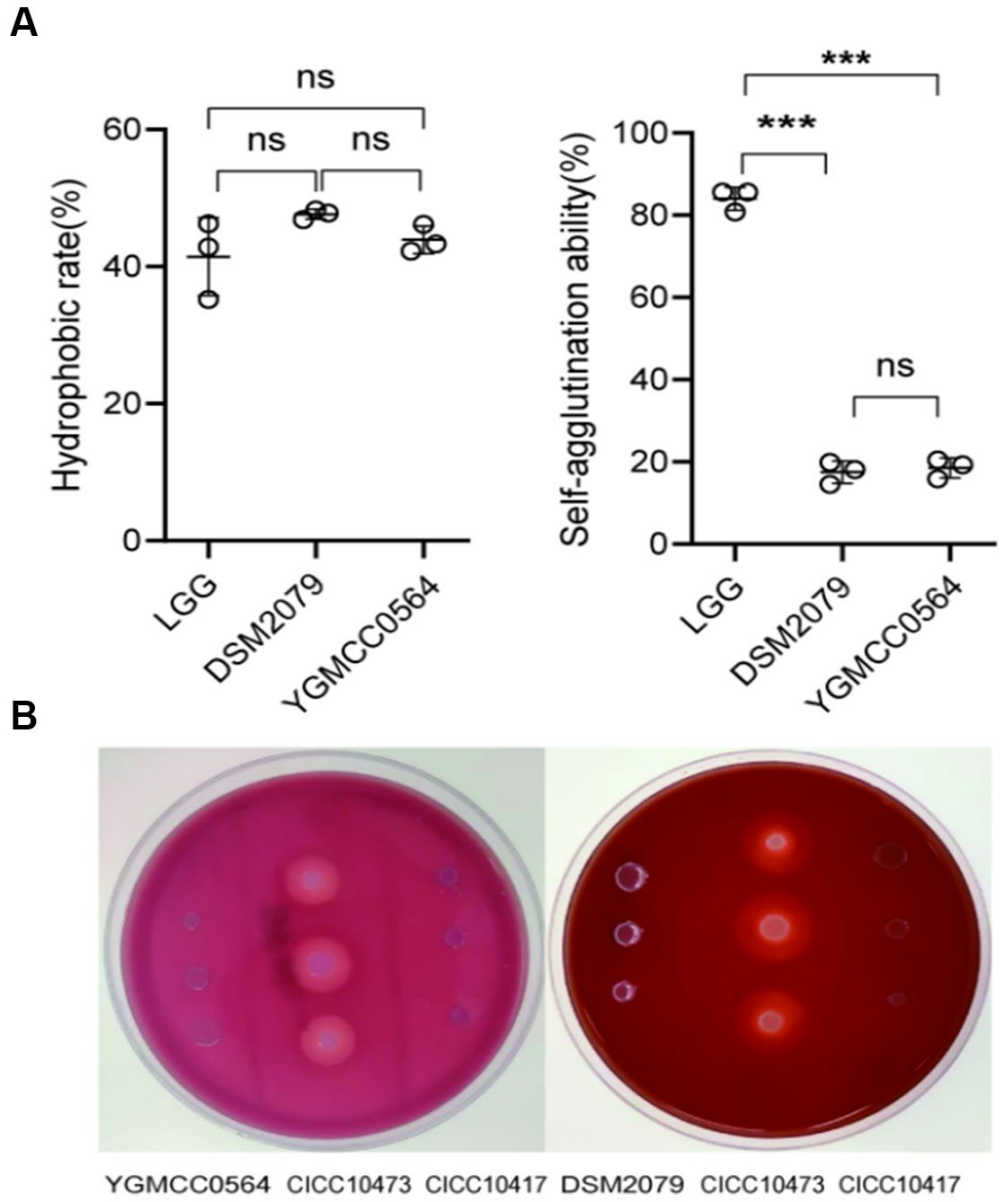
Adhesion and hemolytic activity of YGMCC0564. **(A)** Hydrophobicity and auto-aggregation activity. **(B)** Hemolytic activity of YGMCC0564 and DSM2079. *Staphylococcus aureus CICC 10417* and *Listeria innocua CICC10473* serve as the positive and negative controls, respectively. Error bars represents the SD, student’s unpaired *T* tests, ***p < 0.001, and the independent experiments were repeated twice.

The ability of bacteria to colonize themselves in the gut is an important characteristic of probiotics. It has been observed in previous studies that the ability of probiotics to adhere to intestinal cells and colonize the gut is influenced by the chemical composition and physical properties of their cell surface (de Melo Pereira et al., 2018). Adhesion proteins, such as mucins and fibrinogen-binding proteins, play a significant role in this process (Wu et al., 2022). As shown in Table 4, YGMCC 0564 contained genes that encode various cell-surface proteins, such as lipoprotein signal peptidase (*lspA*, K03101), glyceraldehyde 3-phosphate dehydrogenase (*gpr*, K19265), elongation factor Tu (*tuf*, K02358), triosephosphate isomerase (*tpi*, K01803), encoding for chaperonin (*groeL*, K04077), and co-chaperone (*groeS*, K04078), which likely contribute to its adhesion activity (Yuan et al., 2008; De Angelis et al., 2015; Ye et al., 2020; Sun et al., 2022). Moreover，the hydrophobicity of their cell surfaces plays a role in promoting their ability to adhere to surfaces, and bacteria that aggregate together are the first step in this adhesion process, forming a barrier. The adhesion properties of YGMCC0564 were compared to LGG (positive reference) in Figure 4A. It was found that YGMCC0564 and DSM 2079 had high hydrophobicities of 43.91 and 47.65% in chloroform, respectively. Additionally, LGG had the highest auto-aggregation activity (84.03%), followed by YGMCC0564 and DSM 2079 (Table 4).

**Table 4:** *B. dorei* YGMCC 0564 cell-surface genes.

Name	Number	Function
*lspA*	K03101	Lipoprotein signal peptidase
*gpr*	K19265	Glyceraldehyde 3-phosphate dehydrogenase
*tuf*	K02358	Elongation factor Tu
*tpi*	K01803	Triosephosphate isomerase
*groeL*	K04077	Chaperonin
*groeS*	K04078	Co-chaperone

### Hemolytic test

To evaluate the hemolytic activity of the strains, Columbia blood agar plates were used. In Figure 4B, it was observed that compared to *Staphylococcus aureus* CICC 10473 (positive reference), YGMCC0564 did not form any hydrolysis circles around the colonies, indicating that it is a safe probiotic candidate strain. *Listeria innocua* CICC 10417 was used as a negative control.

### Antibiotic susceptibility and analysis of virulence genes

Whole genome sequencing was conducted to identify antibiotic resistance genes and virulence genes in the genome of YGMCC0564. Two databases, CARD and ResFinder, were used for this analysis. Three antibiotic resistance genes (ErmF, CfxA5, and tetQ) were identified in the genome of YGMCC0564, which are associated with cephamycin (CTT), lincosamide, and tetracycline antibiotics (TE), respectively (Table 5). However, despite the presence of these resistance genes, the susceptibility of YGMCC0564 and DSM2079 to 9 types of antibiotics was shown in Table 6. YGMCC0564 was found to be susceptible to AMS, CTT, IMI, TE, C, and MTZ, but resistant to AMP, MXF, and CD. DSM2079, on the other hand, was susceptible to all antibiotics except for CTT. No potential virulence genes were found in the genome of YGMCC0564, as analyzed using VFDB.3.

**Table 5:** Results of antibiotic resistance genes analysis based on genome sequencing data.

Sample	Gene ID	ARO Name	Drug Class	Identity (%)	Functional description
YGMCC0564	gene2736	*ErmF*	Lincosamide antibiotic	98.5	Antibiotic target alteration
YGMCC0564	Gene3081	*CfxA5*	cephamycin	100	Antibiotic inactivation
YGMCC0564	Gene4103	*tetQ*	Tetracycline antibiotic	98.8	Antibiotic target protection

**Table 6:** Antimicrobial susceptibilities of YGMCC0564.

Number	Name	AMP	AMS	CTT	IMI	TE	MXF	CD	C	MTZ
YGMCC0564	*B. dorei*	R	S	S	S	S	R	R	S	S
DSM2079	*B. thetaiotaomicron*	S	S	R	S	S	S	S	S	S

AMP, Ampicillin; AMS, Ampicillin-sulbactam; CTT, Cefotetan; IMI, Imipenem; TE, Tetracycline; MXF, Moxifloxacin; CD, Clindamycin; C, Chloramphenicol; MTZ, Metronidazole.

### Exploration of the mechanism of cholesterol-lowering effects of YGMCC0564

**Figure 5:**
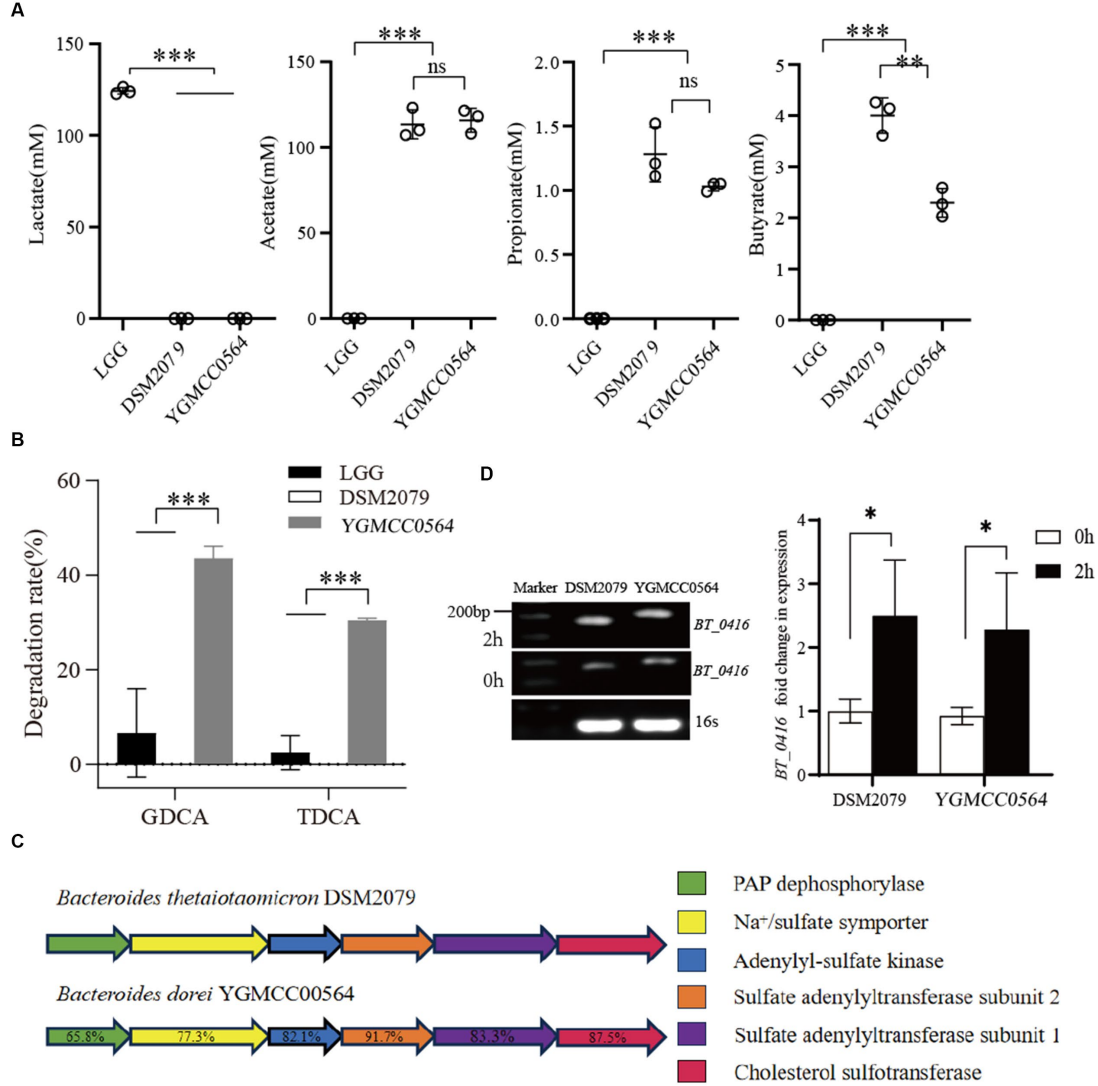
**(A)** Difference in SCFAs produced by YGMCC0564. **(B)** BSH activity of YGMCC0564. Error bars represents the SD, and the independent experiments were repeated twice. **(C)** Cholesterol sulfation biosynthesis gene clusters. **(D)** Expression levels of *BT_0416* genes after 2 h in cholesterol stress. Error bars represents the SD, student’s unpaired *T* tests, *p < 0.05，**p < 0.01，***p < 0.001, and the independent experiments were repeated twice.

HPLC analysis of the supernatants was used to determine the production of lactate, acetate, propionate and butyrate. The levels of SCFAs in the LGG supernatants were significantly different from those of YGMCC0564 and DSM 2079. As shown in Figure 5, LGG was only able to produce a significant amount of lactate (124.31 mM of lactate in MRS medium), but not acetate, propionate or butyrate. However, HPLC analysis confirmed that YGMCC0564, like DSM2079, can produce a certain amount of acetate, propionate and butyrate (Figure 5A).

We also tested the BSH activity of YGMCC0564 using HPLC. The results showed that YGMCC0564 was able to degrade a significant amount of GDCA (43.58%) and TDCA (30.47%), indicating its strong BSH activity. This activity was much higher than that of LGG and DSM 2079 (Figure 5B).

Furthermore, the reduction of cholesterol levels by NGPs involves the crucial role of cholesterol sulfotransferase. DSM2079, a strain highly regulated for cholesterol, harbors *Bt_0416* genes in its genome, and the functionality of these genes has been previously demonstrated (Le et al., 2022).

Our genomic analysis revealed that YGMCC0564 also displays superior cholesterol assimilation potential. By comparing the amino acid sequences of cholesterol sulfotransferase, we identified a homologous cholesterol sulfation biosynthetic gene cluster in YGMCC0564 that resembles DSM2079 (Figure 5C). This gene cluster consists of five key enzymes and their corresponding amino acid homologies: PAP dephosphorylase (65.8%), Na+/sulfate symporter (77.3%), Adenylyl-sulfate kinase (82.1%), Sulfate adenylyltransferase subunit 1 (83.3%), and Cholesterol sulfotransferase *BT_0416* (87.5%, Supplementary Figure S1). These findings suggest that a similar mechanism of cholesterol sulfation biosynthesis exists in YGMCC0564 compared to DSM2079.

To further validate the hypothesis, we assessed the expression levels of the *BT_0416* gene homolog before and after subjecting YGMCC0564 to cholesterol stress, respectively (Figure 5D). Following 2 h of exposure to cholesterol stress, the *BT_0416* homolog exhibited significant up-regulation in YGMCC0564, indicating the involvement of the sulfonated cholesterol gene homolog in YGMCC0564 during cholesterol-induced stress.

## Discussion

In this study, we evaluated the cholesterol conversion efficiency of the isolated *Bacteroides* spp. It was found that out of 66 strains from nine different species, 57 strains (86.4%) had conversion rates between 10 and 20%, suggesting that the conversion ability was fairly consistent among strains, and it was more important to focus on the specific functions of individual strains. We further investigated the strains that had conversion rates above 20% and discovered a highly promising strain called *B. dorei* YGMCC0564, which exhibited high cholesterol degradation, BSH activity, and probiotic properties. YGMCC0564 has the potential to be developed as a probiotic or live biotherapeutics for improving CVD and metabolic syndrome.

Previous studies have provided valuable information about *B. dorei*. Studies showed that *B. dorei* was less abundant in obese individuals and had a protective effect against atherosclerosis, as well as lowering serum lipopolysaccharide activity (Yoshida et al., 2018, 2021). In these studies it was also observed that the combination of *B. dorei* and *B. vulgatus* promoted BCAA catabolism in brown adipose tissue, reduced serum branched chain amino acids (BCAA) levels and had a preventive effect on obesity. Another study demonstrated that *B. dorei* metabolites reduced weight gain and improved serum aspartate transaminase (AST) levels in obese mice (Zhang et al., 2015). These findings suggest that *B. dorei* plays an active role in maintaining lipid metabolism balance, although the precise mechanism of its involvement in lipid regulation is not yet known. In 2007, a study reported that a strain with very high homology (>99.5%) to *B. dorei*, D8, could convert cholesterol to prostanol. Prostanol is not easily absorbed by the human intestine, so this conversion is considered a natural way to lower serum cholesterol levels in humans (Gérard et al., 2007). Additionally, DM2079 has the ability to directly convert cholesterol into sulfated cholesterol in the gut due to the presence of a gene encoding a sulfonase enzyme (Le et al., 2022). In our study, we detected high expression of the *BT_0416* homologous gene in YGMCC0564 after exposure to cholesterol stress, suggesting that a similar pathway to DM2079 may exist in YGMCC0564. However, due to limitations in our assay, we were unable to determine the intermediate and final products of the cholesterol sulfonation modification process. Therefore, further investigation is needed to confirm this conclusion.

YGMCC0564 was found to produce high yields of acetate, propionate and butyrate, but not lactate，which suggests that although they could degrade cholesterol, YGMCC0564 may have completely different mode in lipid regulation from LGG. SCFAs are closely connected to human lipid and glucose metabolism. There is evidence to support that in white adipose tissue, short-chain fatty acids can reduce the intake of fatty acids by inhibiting fat accumulation through the activation of G protein-coupled receptor 43 (GPR43) (Kimura et al., 1829). Additionally, overweight men who received distal acetate infusion experienced an increase in fasting fat oxidation. Furthermore, propionate and butyrate stimulate the production of the satiety hormone leptin in adipocytes by activating free fatty acid receptor 3 (FFAR3) on the cell surface (Xiong et al., 2004). Butyrate alone has been found to reduce visceral and hepatic fat (Chambers et al., 2015). Based on these findings, it is likely that YGMCC0564 exerts lipid-lowering effects *in vivo* through multiple pathways involving SCFAs and cholesterol degradation. However, further research is needed to understand the exact regulation of lipid metabolism *in vivo*.

Probiotics are defined as “live microorganisms that are beneficial to the health of the host when given in sufficient quantities” (FAO/2014) (Hill et al., 2014). In order for probiotics to have a probiotic effect, they need to survive in the human gastrointestinal tract (GIT) and adhere to the intestinal surface (Gut et al., 2018). LGG, a commonly used probiotic, is known for its excellent gastrointestinal tolerance and adhesion to the intestinal epithelium (Capurso, 2019). Liu et al. evaluated the gastrointestinal fluid tolerance of *Lactobacillus* and *Bifidobacterium* based on the reference LGG (Liu et al., 2020). In this study, we compared the gastrointestinal fluid tolerance and adhesion capacity of YGMCC0564 to LGG and other strains. We found that YGMCC0564 showed high tolerance to *in vitro* condition using solutions that simulated gastric and intestinal pH and enzymatic contents, similar to LGG but significantly higher than DSM2079. However, other strains like YG-BU5, YG-BU7, and YG-BU10 did not survive under the same conditions. The adhesion capacity of probiotics to intestinal mucosal surfaces can vary between strains. Therefore, it is important to use a combination of methods to assess the adhesion capacity of probiotics (Monteagudo-Mera et al., 2019). Cellular self-aggregation and surface hydrophobicity are classical methods for assessing the adhesion capacity of probiotics. Cellular self-aggregation forms a barrier against the adhesion of pathogenic bacteria, while surface hydrophobicity enhances adhesion capacity. Compared to LGG, YGMCC0564 exhibited higher cell surface hydrophobicity and its self-aggregation ability was not significantly different from DSM2079.

The numerous studies on probiotics have extensively documented their health benefits. However, with the emergence of new probiotic strains in recent years, it is crucial to assess their safety before they can be consumed and marketed for human use. The International Scientific Association of Probiotics and Prebiotics (ISAPP) has provided recommendations in 2022 on the main methods for evaluating the safety of probiotics. These methods include conducting experiments on antibiotic susceptibility, hemolytic activity, biogenic amine production, potential virulence factors, and *in vivo* oral toxicity tests (Merenstein et al., 2023). The concept of presumption of eligibility for safety, as outlined by the EFSA, emphasizes the need to comprehensively evaluate antibiotic resistance, including resistance genes and antibiotic susceptibility (Brodmann et al., 2017). In our study, we examined the presence of antibiotic resistance genes in the YGMCC0564 and found that they were located on the chromosome, indicating a low risk of transferable antibiotic resistance genes. To comprehensively assess antibiotic resistance, we followed the test criteria of the Clinical and Laboratory Standards Institute (CLSI) (Ahire et al., 2021) and tested YGMCC0564 against nine common types of antibiotics belonging to different drug classes. However, we observed inconsistencies between the predictions of the drug resistance database and the results of our *in vitro* experiments. For example, contrary to the predictions of the CARD and ResFinder databases, YGMCC0564 was found to be sensitive to both CTT and TE. Additionally, YGMCC0564 showed resistance to AMP, even though penicillins did not meet the criteria for significant annotation of resistance genes. Similar to our findings, Hou et al. reported that the database predicted inconsistencies between the resistance genes of AKK strains and their antibiotic susceptibility, indicating strain dependence in antibiotic resistance (Hou et al., 2023). Predictions based solely on antibiotic resistance databases may lead to inaccurate assessments, as genotype and phenotype are both required to determine strain-specific antibiotic resistance. Therefore, the resistance of YGMCC0564 will be further investigated by the following aspects: (i) analyzing the presence and distribution of resistance genes with high-resolution whole-genome mapping; (ii) testing its sensitivity and tolerance to different classes of antibiotics; (ii) evaluating its horizontal and vertical transferability of resistance genes. Moreover, the efficacy and safety of YGMCC0564 in combination with antibiotics will be systematically assessed, along with their synergistic or antagonistic effects on antibiotic treatment and their impacts on the intestinal microbiota and resistome. In our analysis of the YGMCC0564 genome, we also examined its virulence potential by comparing it against a database of virulence factors from various pathogens. No virulence genes were identified for YGMCC0564, indicating a very low virulence potential. Hemolysis is a common virulence factor in pathogens that contributes to microbial access to iron and leads to host anemia and oedema (Elli et al., 2000). Most *Lactobacillus* and *Bifidobacterium* did not have beta-hemolytic potential, but a recent study noted that beta-hemolysis was observed in *Lactobacillus paracasei* L3B21R1 and L3B21R2 (Domingos-Lopes et al., 2017). Therefore, it is important to assess the hemolytic potential of new probiotic strains to ensure they do not cause hemolysis after ingestion. In our study, we observed that YGMCC0564 colonies did not produce a hydrolyzed hyaline ring, indicating no hemolytic activity. Based on these findings, we conclude that *B. dorei* YGMCC0564 is a safe candidate of NGPs. Further data on the safety of YGMCC0564 will be provided through oral toxicity testing in the future.

## Conclusion

Sixty-six *Bacteroides* strains obtained from 35 healthy women fecal samples were evaluated for their potential cholesterol assimilation ability and probiotic properties. Out of these strains, *B. dorei* YGMCC0564 demonstrated remarkable cholesterol removal capability and BSH activity. It also exhibited excellent potential as a probiotic, including high cell adhesion ability, tolerance of simulate digestive tract, antibiotics susceptibility, and no hemolysis or virulence genes. Based on these findings, YGMCC0564 shows promise as a candidate for preventing and treating CVD. However, further *in vivo* and clinical studies are required before considering *B. dorei* YGMCC0564 for use as dietary supplements or live biotherapeutics.

## Data availability statement

The original contributions presented in the study are included in the article/Supplementary material, further inquiries can be directed to the corresponding authors.

## Ethics statement

This study protocol was approved by the Ethics Committee of the Chinese Centre for Disease Control and Prevention (Beijing, China, agreement no. 2023-001) and all volunteers provided written informed consent prior to participation. The studies were conducted in accordance with the local legislation and institutional requirements. Written informed consent for participation in this study was provided by the participants’ legal guardians/next of kin.

## Author contributions

ZH: Data curation, Formal analysis, Investigation, Methodology, Project administration, Supervision, Visualization, Writing – original draft, Writing – review & editing. TW: Data curation, Methodology, Visualization, Writing – review & editing. SZ: Data curation, Visualization, Writing – original draft. KS: Methodology, Visualization, Writing – original draft. FW: Data curation, Methodology, Visualization, Writing – original draft. YL: Resources, Supervision, Writing – review & editing. CL: Funding acquisition, Project administration, Resources, Supervision, Writing – review & editing. JC: Data curation, Funding acquisition, Investigation, Methodology, Project administration, Resources, Software, Supervision, Writing – original draft, Writing – review & editing.
