# Supplementary material for: Evaluation of cholesterol transformation abilities and probiotic properties of Bacteroides dorei YGMCC0564
Source: Front Microbiol. 2023 Nov 9;14:1279996. doi: 10.3389/fmicb.2023.1279996 (PMC10666794; doi:10.3389/fmicb.2023.1279996)
Supplement: Supplementary file 1 [file Data_Sheet_1.docx]

# Supplementary Material

Supplementary Table 1. HPLC analysis of SCFAs

| Parameters | Conditions | | | |
| --- | --- | --- | --- | --- |
| HPLC | Waters Arc Premier HPLC system | | | |
| Column | Xselect HSS T3 column (4.6 × 250 mm, 5 µm) | | | |
| Mobile solvent | Time (min) | Flow rate | A: Acetonitrile (%) | B: Phosphate solution (%) |
|  | 0 | 1 | 5 | 95 |
|  | 8.00 | 2 | 5 | 95 |
|  | 16.00 | 2 | 5 | 95 |
|  | 16.01 | 1 | 5 | 95 |
|  | 20.00 | 1 | 5 | 95 |
| Column temperature | 30℃ | | |  |
| Injection volume | 5 μL | | |  |
| Detector | UV/Vis 210nm | | |  |
| Sampling rate | 10 points/second | | |  |
| Data model | Absorbance - MBF | | |  |

Supplementary Table 2. HPLC analysis of conjugated bile salts

| Parameters | Conditions | | |
| --- | --- | --- | --- |
| HPLC | Waters Arc Premier HPLC system | | |
| Column | Waters TC-18 C18 reversed-phase chromatography column (4.6 × 250 mm, 5 µm) | | |
| Column flow rate | 1 mL/min | | |
| Mobile solvent | A: 7.5mmol/L tetra-butylammonium bisulfate in acetonitrile-water solution (60:40, pH 2.5) | | B: 7.5mmol/L tetra-butylammonium bisulfate in acetonitrile-water solution (30:70, pH 2.5) |
|  |  | 10% | 90% |
|  | The percentage of mobile phase B increases from 10% to 70% in 30 minutes | | |
| Flow rate | 1.5 mL/min | | |
| Column temperature | 30℃ | | |
| Injection volume | 20 μL | | |
| Detection wavelength | 200 nm | | |
| Detector | Waters 2489 UV / visible light | | |

Supplementary Table 3. Primers for qPCR

| Primer | Sequences(5’-3’) |
| --- | --- |
| 16s-F | GGTAGTCCACACAGTAAACGATGAA |
| 16s-R | CCCGTCAAATTCCTTTGAGTTTC |
| *BT_0416*(B.T)-F | CCAGCCTCTGAAACTGGAGG |
| *BT_0416*(B.T)-R | TTCTCCGTCATTCCCATCGC |
| *BT_0416*(B.D)-F | TAGGCGCCGATTGGAAAACT |
| *BT_0416*(B.D)-R | AGTCCCACTACGCCAATGTC |
| *ismA*-F | GGGCTTCACAAAGACAGACA |
| *ismA*-R | GTGCTCAATGCCGAACATAATC |


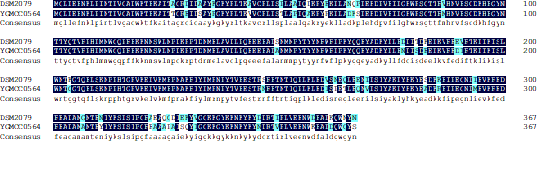


Supplementary figure 1. BT_0416 amino acid sequence alignment. The blue background indicates the conserved amino acid sequence
